# Comparison of blind end closure techniques in equine ileum

**DOI:** 10.1111/vsu.70161

**Published:** 2026-09-22

**Authors:** Rachel L. Hilliard, Amanda Prisk, Sichao Wang, Stefanie Hansen, Amelia Munsterman

**Affiliations:** ^1^ Department of Large Animal Clinical Sciences College of Veterinary Medicine, Michigan State University East Lansing Michigan USA; ^2^ Department of Large Animal Clinical Sciences Cummings School of Veterinary Medicine, Tufts University North Grafton Massachusetts USA; ^3^ Center for Statistical Training and Consulting (CSTAT) Michigan State University East Lansing Michigan USA

## Abstract

**Objective:**

To determine the difference in bursting pressure (BP), bursting wall tension (BWT), and closure speed between commonly utilized methods of ileal stump closure.

**Study design:**

Randomized experimental study.

**Sample population:**

A total of 71 ileal segments from 18 healthy horses.

**Methods:**

Ileum was collected postmortem from 18 horses and divided into four segments. One end of each segment was closed using one of three techniques: Parker‐Kerr (PK), gastrointestinal staples (ST), or gastrointestinal staples with a Lembert staple oversew (SO). Closure time was recorded. Control (C) segments were not altered. Segments were distended until failure. BP and infusion volume were documented, and BWT was calculated.

**Results:**

BP and BWT were higher for controls compared to all closure methods (*p* < .001). No differences in BP (*p* > .999) or BWT (*p* > .77) were found between closure methods. ST technique was 27.1 times faster than PK (*p* < .001) and 23.4 times faster than SO (*p* < .001). SO was 1.2 times faster than PK (*p* < .001). Mechanisms of failure were predictable within each technique.

**Conclusion:**

No difference in BP or BWT was observed between closure techniques in healthy ileum. ST and SO were significantly faster than PK.

**Clinical significance:**

The similarity in bursting strength across techniques suggests that closure method should be chosen based on other factors. The ST closure was faster compared to other techniques, and the addition of an oversewing pattern did not improve security of closure.

**Abbreviations**
BP: bursting pressure
BWT: bursting wall tension
C: control
GLMM: generalized linear mixed model
ICC: intraclass correlation coefficient
IQR: interquartile range, linear mixed model (LMM)
LRT: likelihood ratio test
PK: Parker‐Kerr
ST: stapled
SO: Stapled with oversew
TA: thoracoabdominal
TA 90: thoracoabdominal 90 mm

## 1 INTRODUCTION

Jejunocecostomy is frequently performed in horses with strangulating lesions of the ileum and/or distal jejunum.^1^ Prior to the jejunocecal anastomosis, jejunocecostomy requires creation of a blind‐ended ileal stump aboral to the resection site. Multiple surgical techniques are documented for closure of the ileal stump, including hand‐sutured closure using a Parker Kerr (PK) pattern, closure with gastrointestinal staples, or closure with gastrointestinal staples oversewn with an inverting suture pattern.^2^

The PK suture pattern is commonly used to close hollow viscera, such as the bladder, uterus, or intestine.^3^, ^4^, ^5^, ^6^, ^7^ It allows for resection, inverted closure, and oversew without lumen exposure, decreasing the risk of contamination. Some studies have also shown that hand‐sewn anastomosis has superior bursting strength compared to oversewn stapled anastomosis.^8^

Thoracoabdominal (TA) staplers fire a staggered double row of B‐shaped titanium staples, which seal tissues while preserving microcirculation.^4^ Stapled closure of intestine may offer advantages including decreased surgical time^9^ and higher tensile strength during the healing lag phase.^10^ However, TA staplers are more expensive than the equivalent amount of absorbable suture. TA staples also cause mucosal eversion, which can result in delayed healing, a prolonged inflammatory response, and adhesion formation.^11^, ^12^

Oversew of the everted staple line is one solution to decrease mucosal exposure and subsequent peritoneal contamination. Addition of an oversew has been shown to increase anastomosis security in non‐equid species. In dogs undergoing typhlectomy, cecum closed with staples and oversewn in a Cushing pattern burst at a significantly higher pressure than cecum closed using PK suture or staples alone.^13^ Another retrospective canine study showed that oversew of the staple line was associated with reduced dehiscence during functional end‐to‐end stapled intestinal anastomoses.^14^ When comparing inverting patterns, prior research has shown no difference in bursting pressure (BP) between Cushing and Lembert suture patterns.^15^

Despite multiple studies examining gastrointestinal blind‐end closure security in other species, there is a paucity of analogous research in the equine gastrointestinal tract. In horses undergoing jejunocecostomy, choice of ileal closure technique may be dictated by surgeon preference, speed of closure, availability and cost of stapling equipment, or belief that the pattern will adequately prevent leakage. However, there is little empirical data directing technique for equine ileal stump closure in the current literature. As such, the objective of this study was to determine if a significant difference existed between commonly utilized methods of ileal stump closure in healthy equine intestine based on comparison of BP, bursting wall tension (BWT), and closure speed. We hypothesized that ex vivo ileal segments closed with a PK pattern or gastrointestinal staples with a Lembert staple oversew (SO) would withstand higher BP and BWT than ileum closed with the gastrointestinal staple technique (ST) alone. We also hypothesized that speed of closure would be significant faster for ST and SO compared to PK.

## 2 MATERIALS AND METHODS

Ileum was collected from adult horses (>2 years of age) euthanized for reasons unrelated to gastrointestinal pathology. Prior to starting the study, a power analysis was performed which indicated that a sample size of 18 horses would give a target power of 0.75 and type 1 error/alpha value of 0.1. Exclusion criteria included known recent gastrointestinal disease, recent treatment with phenylbutazone or corticosteroids, and clinical evidence of or known diagnosis with pars pituitary intermedia disease. Horses over 25 years of age, draft/pony breeds, and subjects with gross ileal abnormalities on necropsy were also excluded. All subjects were donated from private owners after euthanasia or euthanized for other research purposes, and written consent was obtained to use tissues in adherence to the institutional research standards.

Within 1 h of euthanasia, sections of ileum extending from the ileocecal junction to the first jejunal arcade were collected via ventral midline incision, rinsed with water, and placed in isotonic saline solution. Samples were refrigerated at 4^o^C for up to 6 h prior to testing. At the time of testing, each ileum was divided into four segments of equal length. A randomized block design using computer generated numbers (random.org) was used to assign the segments from each horse into four groups: control (C), PK, ST, or SO. The aboral end of the first and third segments and the oral end of the second and fourth segments were closed using the assigned technique, and control segments were left open. All closures were performed by the same ACVS surgical resident (RLH) under supervision of a board‐certified large animal surgeon.

For the PK technique, the end of the ileal segment was clamped with Rochester‐Carmalt forceps. The protruding ileum was trimmed with Mayo scissors, leaving 2–3 mm adjacent to the forceps. The intestine was then sutured with 2–0 glycomer 631 on a taper needle (Biosyn, Medtronic, Minneapolis, Minnesota) in a Cushing pattern over the forceps, leaving the tail end untied. Each suture bite was approximately 6 mm long and placed approximately 3 mm from the forceps. The forceps were removed and the suture was pulled from both ends to invert the mucosa. The leading end of the suture was then used to perform a Lembert oversew, starting 8 mm from the inverted edge and emerging 3 mm from the inverted edge, with bites approximately 4 mm apart. The last bite of the Lembert pattern was tied to the tail of the first bite, in a surgeon's knot followed by five additional throws (Figure 1A). “Closure time” was measured from the start of the first bite until the completion of the final knot.

**FIGURE 1:**
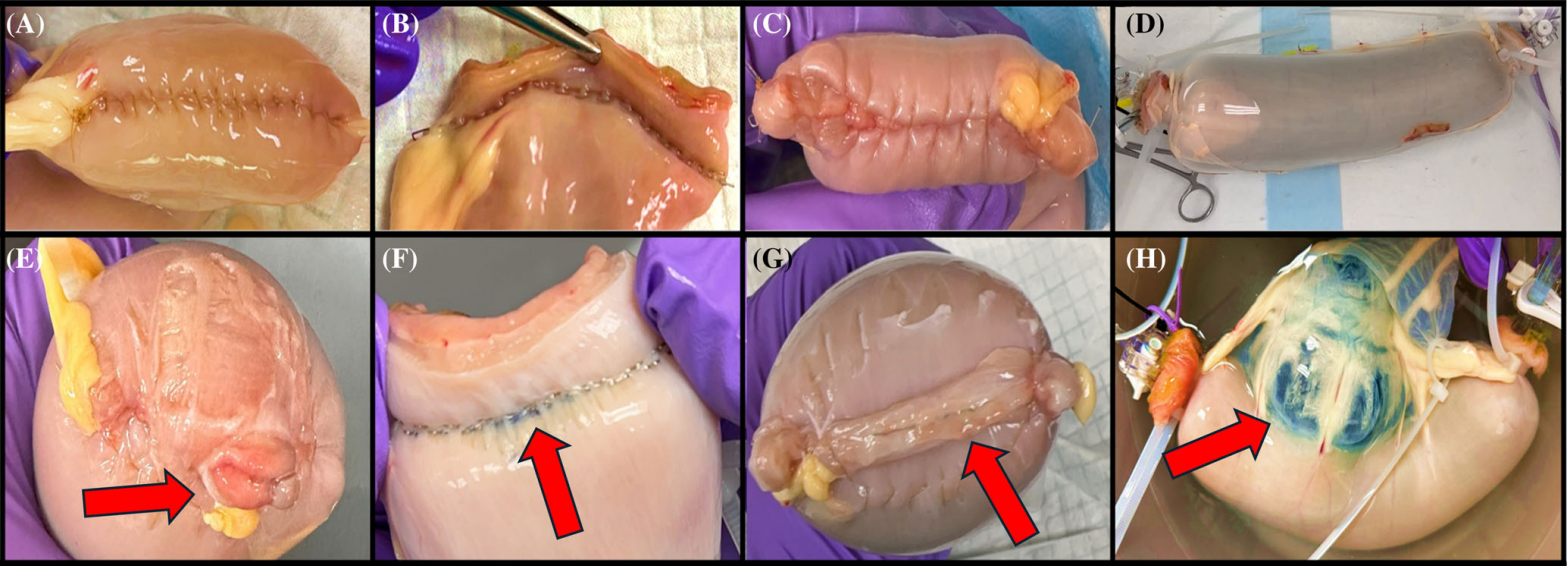
Examples of closed ileal segments (top row) and the corresponding segment following distension until failure (bottom row). Mode of failure was consistent and specific to each closure technique. Parker‐Kerr (A) most often failed by pull‐through of the oversew first, followed by rupture through the inner suture line (E, red arrow). Stapled closure (B) most often failed by leakage between staples (F, red arrow indicating leakage). Stapled closure with Lembert oversew (C) most often failed by oversew pull‐through, then leakage between staples (G, red arrow indicating leakage). Control segments (D) usually failed via dramatic rupture at the mesenteric edge (H, red arrow).

For the ST technique, a thoracoabdominal 90 mm (TA 90) reusable stapling device (Covidien, Mansfield, Massachusetts) with 4.8 mm long, 4 mm wide, B‐shaped staples was used. The device was clamped onto the end of the ileal segment, and the segment was trimmed with Mayo scissors to leave approximately 2–3 mm adjacent to the stapler (Figure 1B). “Closure time” was measured from just prior to clamping the stapler to release of the stapling device.

For the SO technique, the TA 90 reusable stapling device was applied as described above. The staple line was then oversewn with 2–0 glycomer 631 on a taper needle in a Lembert pattern, starting approximately 8 mm from the staple line and emerging 3 mm from the staple line, with bites 4 mm apart (Figure 1C). Knots at the start and end of the suture line were created with a surgeon's knot followed by five additional throws. “Closure time” was measured from just prior to clamping the stapling device to the final knot of the Lembert pattern.

For mechanical testing, a pressure sensor (SPR‐350S, Millar Instruments, Inc., Houston, Texas) was placed using a Touhy‐Borst adapter and three‐way stopcock and sealed in the open end of each ileal segment with two 20 cm cable ties (Hyper Tough, Walmart, Bentonville, Arkansas). A fluid line was attached to the stopcock and the ileum was placed in a water bath. For control segments, the opposite end was also sealed with a closed three‐way stopcock and two cable ties (Figure 2). A calibrated peristaltic infusion pump was used to distend the intestinal segments at 1000 mL/min with distilled water tinted with new methylene blue (Ricca Chemical Company, LLC., Arlington, Texas) until failure was observed by leakage of the dye or catastrophic rupture.

**FIGURE 2:**
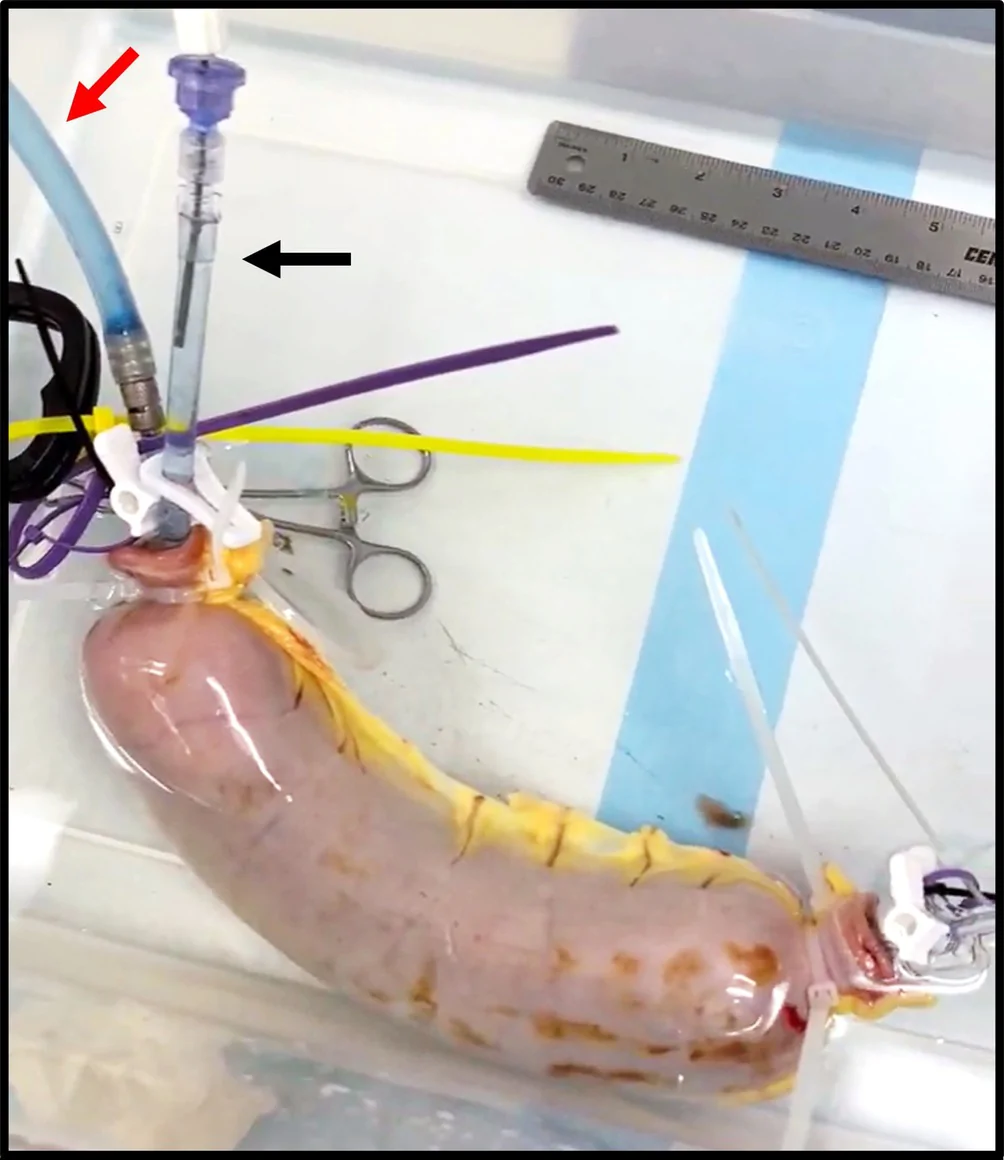
Example of mechanical testing unit for measurement of ileal bursting pressure. In this image, a control ileal segment is suspended in a water bath, with both ends sealed with cable ties. A piezoelectric pressure sensor (black arrow) is sealed with a Touhy‐Borst adapter within one side of the intestine. A fluid line (red arrow) connected to a peristaltic infusion pump is attached to a three‐way stopcock sealed within the same end of the ileum.

Maximum BP, indicated as the ultimate pressure prior to catastrophic failure or leakage of the dye, and total volume infused were recorded. Videos of each test were also recorded, with a calibrated measuring device placed adjacent to the intestinal segment for post hoc measurement. BWT was calculated as previously described.^16^ In short, BWT was calculated by LaPlace's law (BWT = BP × radius at failure). Radius (*r*) at the time of failure was calculated by the formula *r* = √ (volume/*π* × length). Volume (*V*) was measured as the volume of water instilled in milliliters, calculated from the weight of the fluid bag prior to distension and immediately after failure. Length (*l*) was calculated from still images of the ileal segments taken from the video at the moment prior to failure. All image measurements were obtained in triplicate by three separate observers (RLH, AM, AP) using commercial software^17^ (ImageJ, National Institutes of Health) and averaged for the mean.

### 2.1 Statistical analysis

Ileal length, BP, and BWT are reported as mean ± SD. To account for repeated measurements, a linear mixed model (LMM) was used for these variables, with “horse” as a random intercept and “ileal stump closure method” and “segment location” (oral vs. aboral) included as fixed factors. The interaction term between ileal stump closure method and segment location was removed from final models based on the likelihood ratio test (LRT). Closure time is reported as median with interquartile range (IQR). Because closure time data were non‐normally distributed, a generalized linear mixed model (GLMM) with a gamma distribution and log‐link function was employed, utilizing “horse” as a random intercept and “closure method” as a fixed factor.

For all models, pairwise comparisons among methods were performed with Holm‐Bonferroni adjustments for *p*‐values. Model assumptions were verified by visual inspection of conditional residual plots. Intra‐ and interrater reliability were estimated using intraclass correlation coefficients (ICC) based on a two‐way mixed‐effects model for absolute agreement. Statistical analyses were performed using R (version 4.5.0)^18^ with the lme4, glmmTMB and irr packages.^19^, ^20^, ^21^

## 3 RESULTS

A total of 18 horses were enrolled in the study. Breeds included Thoroughbred (7), Quarter Horse (5), Arabian (2), Standardbred (2), Appaloosa (1), and stock‐type cross (1). A total of 13 geldings and five mares were enrolled. The median weight of enrolled horses was 490.5 kg (range: 398 kg–631 kg). Median subject age was 11 years (range: 3–22 years).

Total harvested ileal length ranged from 60 cm to 96 cm, with a median of 70 cm. One control segment was excluded from analysis due to cable tie dysfunction during testing. One SO segment was excluded from closure time analysis due to timer malfunction.

Failure occurred with a predictable pattern specific to each closure technique (Figure 1E–H). A total of 10/18 (55.6%) of PK segments failed by oversew pull‐out through the seromuscular layer, then rupture of the mucosa/submucosa through the inner suture line. Rupture through the inner suture line commonly occurred near the center of the closure (8/18; 44.4%). Failure of ST segments occurred by leakage between staples in 14/18 (77.8%) cases. Failure of the SO group most often occurred by oversew pull‐out through the seromuscular layer, then leakage between staples (10/18; 55.6%). The control segments failed most often by rupture at the mesenteric edge (12/18; 66.7%).

Measured mean ileal length at maximum distension was 23.83 ± 5.26 cm for control segments, 23.48 ± 4.78 cm for PK, 23.74 ± 3.89 cm for ST, and 25.32 ± 7.12 for SO. Ileal length did not differ between groups (*p* = .483). Intrarater reliability of ileal length measurements was excellent for RLH (ICC = 0.989, 95% CI: 0.984, 0.993), AM (ICC = 0.999, 95% CI: 0.999, 1.0), and AP (ICC = 1.0, 95% CI: 1.0, 1.0). Reliability was also excellent between raters, with an inter‐rater ICC of 0.987 (95% CI: 0.980, 0.991).

Recorded mean BP was 375.6 ± 92.5 mmHg for control segments, 310.1 ± 91.4 mmHg for PK, 296.1 ± 69 mmHg for ST, and 304.2 ± 86.9 mmHg for SO (Figure 3). Segment location had an influence on BP (*p* = .023), although this effect did not remain significant in pairwise comparisons (*p* ≥ .137). Pairwise comparisons of closure technique after adjusting for segment location showed that control BPs were greater than PK, ST, and SO segments (*p* < .001). However, no differences in BP were observed between closure techniques (*p* > .999).

**FIGURE 3:**
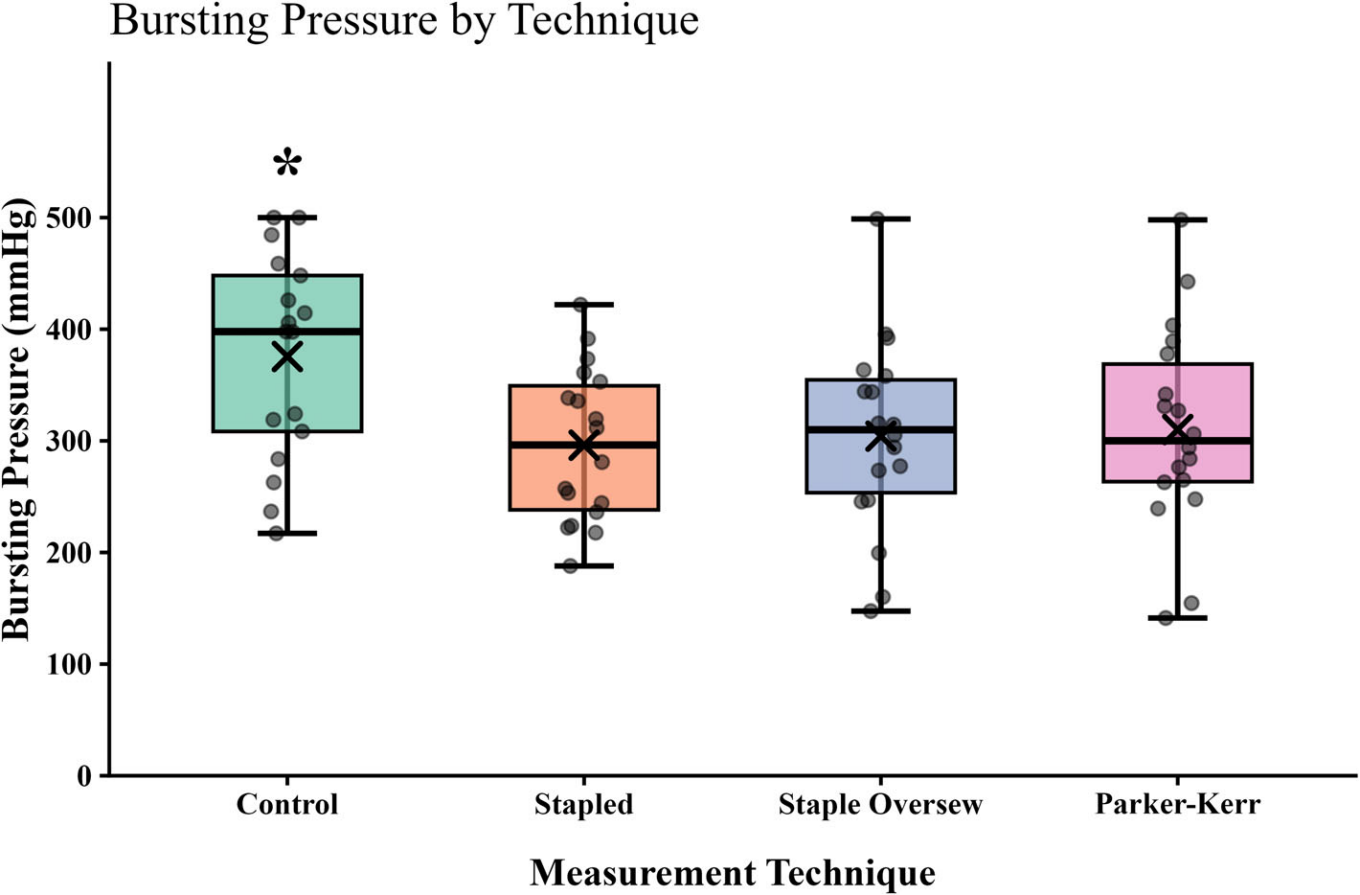
Box plot showing bursting pressure (BP) for control (green), stapled (orange), staple with Lembert oversew (blue) and Parker‐Kerr (pink) closure techniques. Closure technique is on the X‐axis and BP (mmHg) is on the Y‐axis. The box boundaries define the first and third quartiles (*Q*_1_ and *Q*_3_), with the bold center line representing the median. The “*X*” marker denotes the mean. The extended whiskers represent the data range within Q_1_–1.5 × IQR and Q_3_–1.5 × IQR, where IQR is the interquartile range. Significant differences are indicated by the black star. Control BP was higher than all closure methods (*p* < .001). No differences in BP were observed between closure techniques (*p* > .999).

Calculated mean BWT was 1193 ± 322 dynes/cm for control segments, 894 ± 270 dynes/cm for PK, 937 ± 217 dynes/cm for ST, and 917 ± 252 dynes/cm for SO (Figure 4). Segment location also had an effect on BWT (*p* = .018). Pairwise comparison showed that the second from oral segment had a lower BWT at failure than the most aboral segment (*p* = .031). No other segment locations showed differences in BWT. Pairwise comparisons after adjusting for segment location showed that control segment BWT was higher than PK, ST, and SO segments (*p* < .001). No differences in BWT were observed between closure techniques (*p* > .77).

**FIGURE 4:**
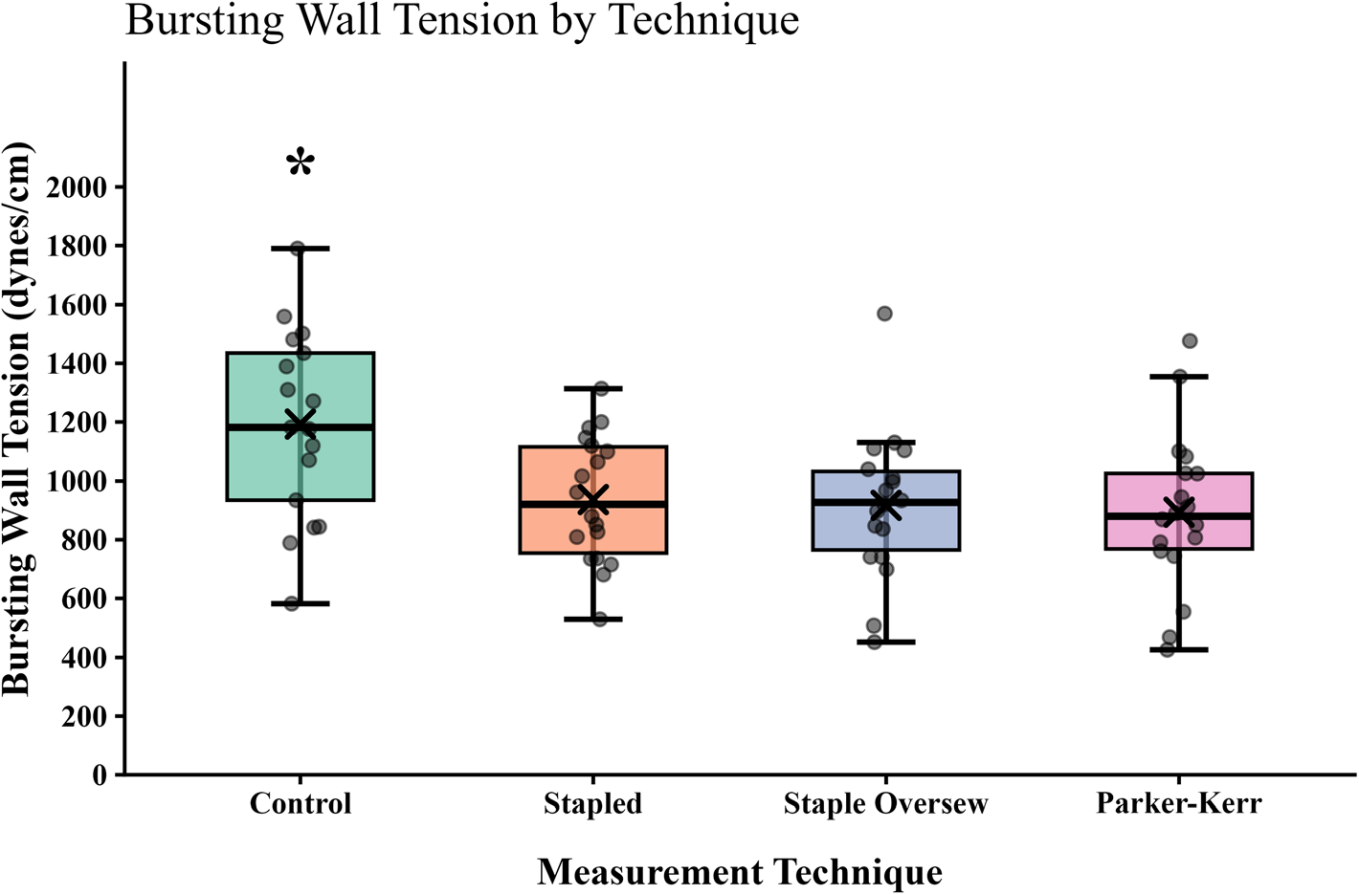
Box plot showing bursting wall tension (BWT) for control (green), stapled (orange), staple with Lembert oversew (blue) and Parker‐Kerr (pink) closure techniques. Closure technique is on the X‐axis and BWT (dynes/cm) is on the Y‐axis. The box boundaries define the first and third quartiles (*Q*_1_ and *Q*_3_), with the bold center line representing the median. The “*X*” marker denotes the mean. The extended whiskers represent the data range within Q_1_–1.5 × IQR and Q_3_–1.5 × IQR, where IQR is the interquartile range. Significant differences are indicated by the black star. Control BWT was higher than all closure methods (*p* < .001). No differences in BWT were observed between closure techniques (*p* > .77).

Recorded median closure time was 973.9 s (IQR: 869.0–1106.4 s) for PK, 34.6 s (IQR: 27.8–44.2 s) for ST, and 834.9 s (IQR: 794.3–943.7 s) for SO (Figure 5). Gamma model comparisons showed that the ST technique was 27.1 times faster than PK (*p* < .001) and 23.4 times faster than SO (*p* < .001). SO was 1.2 times faster than PK (*p* < .001).

**FIGURE 5:**
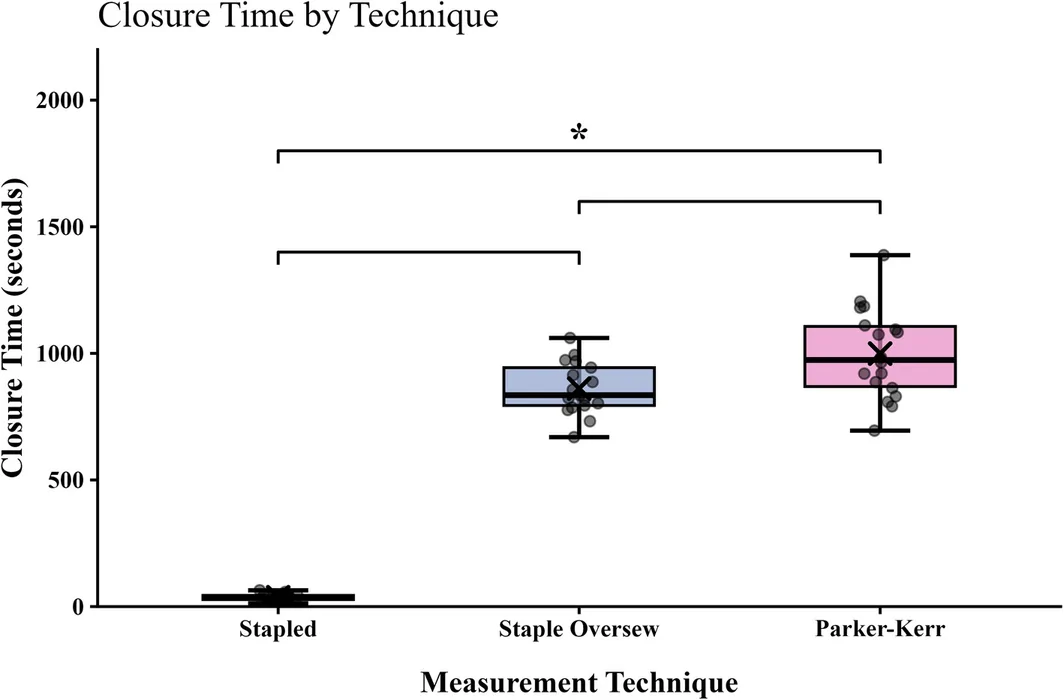
Box plot showing closure time for stapled (orange), staple with Lembert oversew (blue) and Parker‐Kerr (pink) closure techniques. Closure technique is on the X‐axis and closure time (seconds) is on the Y‐axis. The box boundaries define the first and third quartiles (*Q*_1_ and *Q*_3_), with the bold center line representing the median. The “X” marker denotes the mean. The extended whiskers represent the data range within *Q*_1_–1.5 × IQR and *Q*_3_–1.5 × IQR, where IQR is the interquartile range. Significant differences are indicated by the black star and horizontal bars. Stapled closure was 27.1 times faster than Parker‐Kerr (*p* < .001) and 23.4 times faster than stapled oversew (*p* < .001) techniques. Staple oversew was 1.2 times faster than Parker‐Kerr (*p* < .001).

## 4 DISCUSSION

The results of this study did not support the first hypothesis, showing no difference in BP or BWT between ileal segments closed with PK, ST, or SO techniques. All techniques demonstrated lower bursting strength than control segments, indicating loss of integrity in tissues closed by sutures or staples. The second hypothesis was supported, as both stapled closures were faster than the Parker Kerr technique.

Although resection of the proximal ileum during jejunocecostomy eliminates normograde flow of ingesta, leakage of the ileal stump has been a documented cause for repeat celiotomy and euthanasia post‐jejunocecostomy.^22^, ^23^, ^24^, ^25^, ^26^ In this study, we showed that none of the evaluated closure patterns displayed an advantage in closure security in healthy intestine. The recorded BPs in all groups were markedly higher than previously documented pressures within the normal distal ileum during cecal reflux (approximately 16.7 ± 4.5 mmHg).^27^ We acknowledge that other factors, such as mural edema and ileal necrosis, are more likely to contribute to ileal stump leakage post‐jejunocecostomy. While these variables are challenging to replicate within a controlled environment, measuring BP and BWT provides an analog to isolate and compare stability of closure techniques. Our findings show equivalence of BP and BWT using all three methods, which suggests that closure technique should be chosen based on factors other than pattern security.

Eversion of intestinal mucosa increases the risk of abdominal contamination and delayed mucosal healing, leading to inflammation, adhesions, and peritonitis.^24^, ^25^ However, prior research shows no significant increase in peritonitis for horses undergoing pelvic flexure closure with TA 90 staples.^28^ Conflicting results exist about whether oversew of stapled blind ends affects the risk of adhesions.^25^, ^29^ It has also been suggested that oversewing everted mucosa may entrap contamination, leading to microabscess formation.^30^ However, this was not evident in one in vivo study examining staple oversew closure of equine jejunum and ileum during jejunocecostomy, which found inflammatory changes but not overt abscessation between the staple line and oversew at 7 days post‐operation.^31^

Conversely, blind ST closure without the addition of an oversewing suture line may be useful to resect a larger portion of devitalized ileum deeper in the abdomen,^2^, ^24^ decreasing overall peritoneal contamination. In these cases, the benefits of removing devitalized bowel and decreased surgical time may outweigh the risks of mucosal eversion. However, caution is warranted when utilizing this technique with severely compromised ileum, as the proinflammatory environment created by strangulating lesions may influence closure viability and adhesion formation. As this study did not seek to quantify degree of bacterial contamination caused by the everted ileal mucosa, further research is necessary to evaluate in vivo inflammatory responses and adverse effects associated with these techniques.

Prolonged surgical duration is a risk factor for surgical site infection in exploratory laparotomy.^32^ ST has a distinct time advantage that may merit consideration when deciding how to close the ileal stump, particularly in severely endotoxic patients and/or those with cardiovascular or pulmonary compromise under anesthesia. The SO pattern was also slightly faster to perform than PK. This conflicts with previous studies, which showed that PK was faster to perform than SO.^31^ It is likely that the difference in closure speed between PK and SO may vary depending on surgeon skill and individual familiarity with the closure pattern. In this study, the difference in median closure time between the two patterns was minimal (2 min, 19 s). This small reduction is unlikely to be clinically relevant when compared to the duration of the entire anesthetic event.

In this study, both sutured techniques failed most often via initial pull‐out of the seromuscular oversew, then failure of the deep layer at pressures similar to that of the ST closure. This suggests that addition of an oversew to gastrointestinal staples in healthy equine ileum does not improve closure strength. Leakage may also be affected by tissue edema and necrosis due to ischemia or pressure, which was not evaluated in this study.

This study has limitations, primarily based on its ex vivo nature. To achieve consistency between samples, healthy ileal segments were used due to the inability to standardize the level of ischemic injury in pathologic ileum. However, in clinical cases the ileum is often severely inflamed, edematous, or necrotic. While it is assumed that friable tissue will respond with a decrease in tissue strength, it is possible that the efficacy of certain closure methods may be more variable in ischemic tissue. For example, the authors found that mucosal inversion during SO closure was more challenging than inversion during PK closure. SO subjectively required more manipulation of the staple line with DeBakey forceps to achieve adequate inversion, which could result in excessive tearing in cases with friable ileal tissue. Stapled patterns are also limited by the width of the stapling device and the ability to adequately compress the tissue between the jaws. TA 90 staples compress to a height of 2 mm,^2^ which may not be feasible in severely edematous tissue. Under these circumstances, the increased flexibility in bite size of a hand‐sewn pattern may be necessary.

In addition, this study could not compare the degree of contamination or inflammation elicited from each closure technique. Comparison of biologic response between inverted and non‐inverted closures remains a necessary avenue for future research.

Ileum harvested during this study extended from the ileocecal junction to the first jejunal arcade. The exact transition from jejunum to ileal is poorly defined. By extending our collected segment to the edge of the first jejunal arcade, it is possible that a portion of thinner‐walled jejunum may have been included in the tested segments. While statistical analysis showed that segment location did influence BP and BWT, it did not influence the overall trend between techniques. Segment location was adjusted for in the final statistical model to remove it as a confounding factor.

Finally, all closures in this study were performed by a single ACVS resident (RLH). Surgeon experience with the suture patterns may affect closure speed. Time spent opening, loading, and positioning the TA90 stapling device was also not evaluated in this study, which may contribute to increased closure time for surgeons less familiar with its use.

## 5 CONCLUSIONS

The results of this study show that there is no difference in BP and BWT between healthy ileal segments closed using PK, ST, or SO techniques. The similarity in bursting strength across patterns suggests that closure method should be chosen based on other factors, such as location of resection or viability of tissues involved in the closure. The ST closure was faster compared to other techniques, providing support for its use. However, further research is necessary to determine the optimal technique in ischemic tissue, as well as to evaluate the clinical response to differences in contamination and inflammation between patterns.

## AUTHOR CONTRIBUTIONS

Hilliard R, DVM: Contributed to experimental design, data acquisition, analysis, and interpretation, literature review, and drafting of the manuscript. Prisk A, DVM, DACVS (Large Animal): Contributed to experimental design, data acquisition, literature review, and editing of the manuscript. Wang S, PhD: Contributed to experimental design, data interpretation, and drafting of the manuscript. Hansen S, DVM, MSc, DACVS (Large Animal): Contributed to data acquisition and editing of the manuscript. Munsterman A, DVM, MS, PhD, DACVS (Large Animal), DACVECC: Contributed to experimental design, data acquisition, analysis, and interpretation, and editing of the manuscript. All authors have read and approved the final version of this manuscript. Additional thanks to Gilday R. DVM, DACVS (Large Animal) for assistance with data acquisition.

## FUNDING INFORMATION

This research was supported by funding from the Tufts Cummings School of Veterinary Medicine Department of Clinical Sciences Faculty Development Fund.

## CONFLICT OF INTEREST STATEMENT

The authors have no conflicts of interest to declare.

## Data Availability

The data that support the findings of this study are available from the corresponding author upon reasonable request.
